# Parameters influencing the fracture of Mo films and their wider significance

**DOI:** 10.1557/s43580-023-00612-3

**Published:** 2023-07-18

**Authors:** M. J. Cordill, P. Kreiml, T. Jörg, S. Zak, C. Mitterer

**Affiliations:** 1 Erich Schmid Institute of Materials Science, Austrian Academy of Sciences, Jahnstrasse 12, 8700 Leoben, Austria; 2 Department of Materials Science, Montanuniversität Leoben, Franz-Josef-Strasse 18, 8700 Leoben, Austria

## Abstract

Fragmentation testing has been used for decades to assess thin film fracture and delamination. Hooke’s law is generally used to determine a film fracture stress from the crack onset strain observed in micrographs or measured as an electrical resistance increase. While this method is in theory suitable in the elastic regime, it neglects important film characteristics, such as residual stress, microstructure, or film architecture. Thus, there is a need to improve fracture analysis using fragmentation to avoid significant errors in measuring fracture stress or apparent fracture toughness of thin films. In-situ X-ray diffraction fragmentation experiments can measure the film fracture stress even for individual layers being part of a multilayer. Which characteristics influence the apparent fracture behavior will be demonstrated on Mo thin films on polyimide.

**Graphical abstract:**

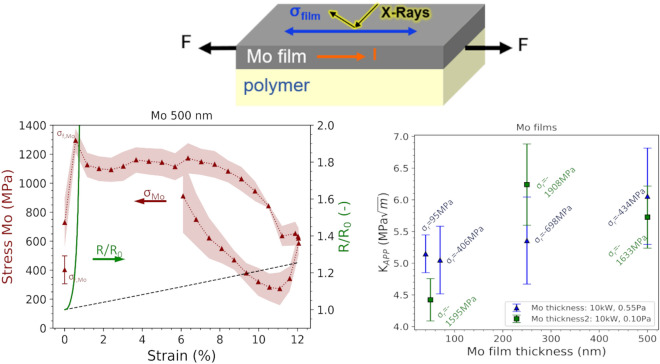

## Introduction

For about two decades, uniaxial tensile testing of stiff films on compliant substrates has been used to evaluate the mechanical and electrical behavior of thin films. The method, also known as fragmentation testing, has several variations with respect to what in-situ parameter is recorded [1–8]. The most common in-situ behaviors to record are the electrical resistance and mechanical damage, or film fracture, with images of the film surface using optical light microscopy [4], confocal laser scanning microscopy (CLSM) [8], atomic force microscopy (AFM) [9], or scanning electron microscopy (SEM) [10]. From the electrical resistance variation, generally described by the constant volume approximation [11, 12], and surface images, the initial fracture strain, *ε*_*f*_, or crack onset strain (COS), can be characterized. When CLSM or AFM methods are used, the adhesion of the film to the substrate could also be evaluated using any delaminations, or buckles, that might form between the cracks [13]. Additionally, after the experiment, the saturation crack spacing, *λ*, can be measured from any suitable images made of the fractured film surface.

With the appropriate inputs, the interfacial shear stress, *τ*_*IFSS*_, or the apparent fracture toughness, *K*_*app*_, of a film can be calculated [1, 14–23]. The *τ*_*IFSS*_ indicates the amount of shear stress that an interface can withstand before fracture and the *K*_*app*_ is a quantitative way to define a film’s resistance to through thickness crack propagation. However, one of the inputs for both factors is the film fracture stress, *σ*_*f*_. Often it is difficult to accurately measure the real fracture stress of a thin film without advanced techniques, such as in-situ straining with X-ray diffraction (XRD), and Hooke’s law is employed with knowledge of the COS (${\sigma }_{f}={\varepsilon }_{f}{E}_{f}$, with *E*_*f*_ the elastic modulus of the film) to obtain apparent fracture stress instead. It has not yet been shown if the use of Hooke’s law is an appropriate alternative way to evaluate the fracture stress of a film under tensile strain since access to the ideal measurement set-ups can be challenging. In-situ straining with XRD is the only method to measure film-only mechanical behavior (for example, the fracture stress) [6, 24–26] and of multiple layers simultaneously [27–29], making it a very powerful technique.

Generally, there are four factors that can affect the fracture stress of thin films on compliant substrates: film thickness, residual stress, microstructure, and film architecture (single versus multilayers) [2]. These factors are also related to the film deposition method and used parameters. The film thickness and residual stress are known to influence the COS and crack spacing for several different film-substrate systems [4, 6, 13, 22, 30–34] with thinner films and compressive residual stresses increasing the COS. The residual stresses of films grown by sputter deposition at room temperature are mainly controlled by the power applied to the magnetron cathodes and/or the process gas pressure used [35]. Microstructure can influence the crack path and, in general, lead to straight (smaller grains) or more wavy cracks (larger grains) compared to the film thickness [36] and influence the amount of plastic deformation before fracture. Crack deflecting microstructures, such as the zig-zag film structure in Mo films [37], have also shown to increase the COS. The film architecture (multilayers and layer order) has been recently examined with Cu/Mo bilayers (Mo as the interlayer) [17] and found that with increasing Cu thickness, the Mo fracture stress also increased to a certain point. It should be noted that the fracture stresses in these various studies were measured differently for each system and do not allow for a direct comparison. While the above examples focus more on Mo thin film systems, similar trends arise in fracture stress measurements for other metal films [19, 23, 25, 30] and oxide films [38–40].

The aim of this study is to use one method to measure fracture stresses of uniaxially strained films to determine how the fracture stresses are influenced to improve the design of thin films for applications in flexible electronics or barrier coatings for food packaging and longevity. Therefore, available in-situ XRD fragmentation data, from both published and unpublished results, for a model material system of sputtered Mo films on Polyimide (PI) will be reviewed and compared. With the new studies and experimental data available, one should be able to describe more accurately how the interfacial shear stress and/or fracture toughness of films are influenced by the film parameters.

## Experimental

All of the Mo thin films used in this study were sputter deposited with either an industrial or a laboratory scale sputtering system on 50 µm thick Upilex PI. The films were deposited between 2014 and 2020 without any additional substrate heating using the same type and manufacturer of Mo target (rotary for industrial and planar for laboratory scale), the Al (industrial) and the Cu (laboratory) targets (both planar) using various deposition parameters. The main parameters that were adjusted were: power, Ar pressure, deposition time (for thickness), and distance to substrate (industrial vs. laboratory scale). For more specifics of the samples, the reader is referred to the following references [17, 32, 35, 37, 41].

**Fig. 1:**
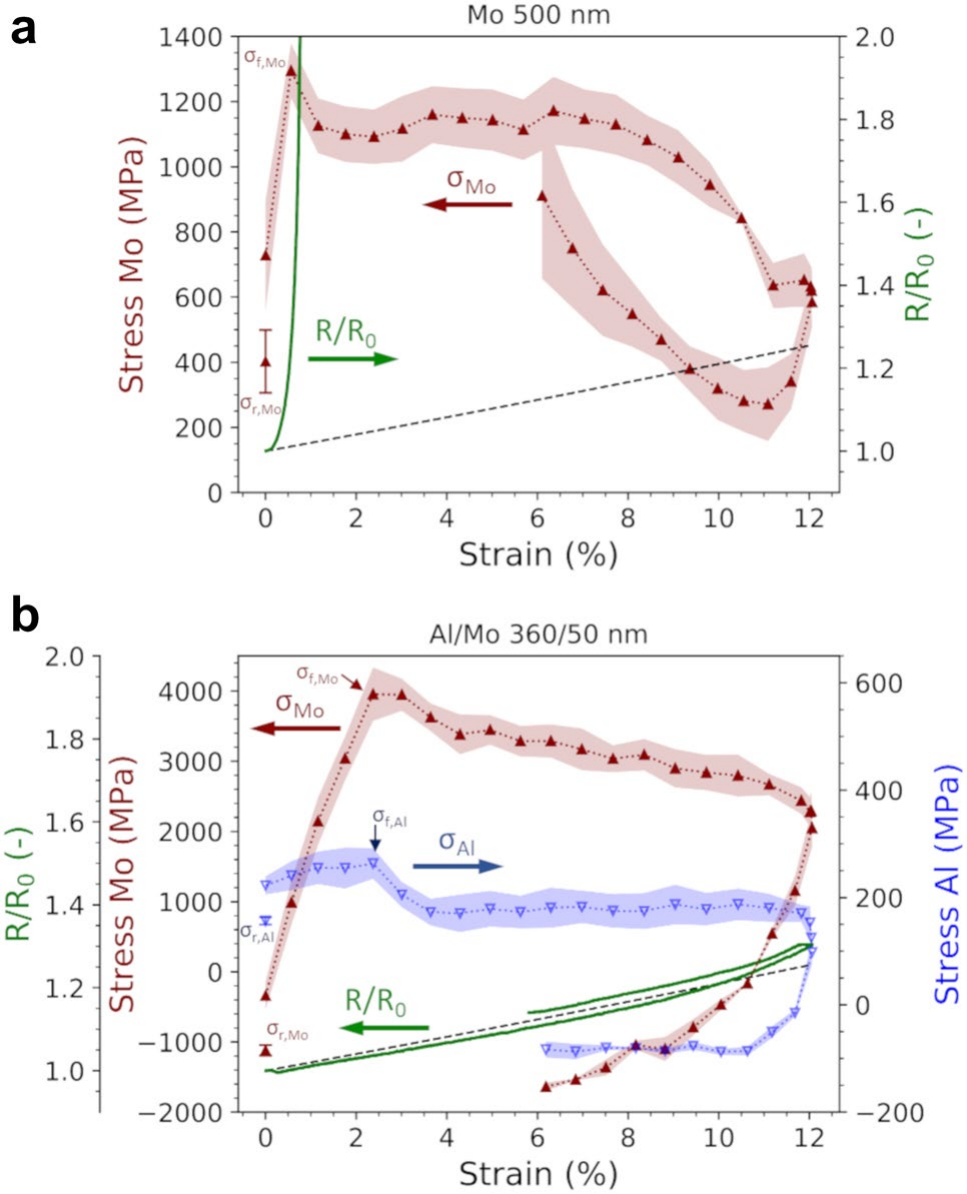
Example film stress–strain curves for **a** single 500 nm Mo film on PI and **b** Al/Mo bilayer (360/50 nm) on PI. The fracture stress and residual stresses for the Mo and Al films are indicated. The dashed line is the constant volume approximation for electrical resistance

In-situ uniaxial straining experiments with XRD and 4-point-probe electrical resistance measurements were performed at the synchrotron beamline KMC-2, BESSY II, at Helmholtz-Zentrum Berlin, Germany [42]. Samples were strained to between 12 and 15% engineering strain while continuously measuring the electrical resistance and collecting XRD patterns for the Mo 110 peak using a Bruker VÅNTEC 2000 area detector and a beam wavelength of 0.154 nm. For the bilayer experiments, the Cu 111 peak or Al 111 peak were collected simultaneously with the Mo films (Mo 110 peak). Five different ψ angles between 0 and 50 degrees were measured consecutively using a 5 s exposure time to use with the sin^2^*ψ* method [43] to measure the lattice strains during straining. A Pearson fit was used to determine the peak positions and widths. The Mo, Cu, and Al film stresses were calculated using X-ray elastic constants (XECs) (1/2 S_2_) [44] for untextured 111 Cu, Al and 110 Mo reflections. XECs were calculated from single-crystal elastic constants assuming the Hill model with the software ElastiX [45]. An example of stress–strain curves measured for a single Mo film and an Al/Mo bilayer are shown in Fig. 1 as well as how COS, fracture stress, and the residual stresses are evaluated. The Mo film stress–strain curves are characteristic of brittle film behavior as the stresses initially increase to a maximum value (peak stress or fracture stress) before an immediate stress decrease that signals film fracture and crack formation.

The general theory behind fragmentation testing is the shear lag model [1, 46, 47] that can be used to determine the interfacial shear stress, *τ*_*IFSS*_, to quantify the stress the interface can carry (Eq. 1):

1 $${\tau }_{IFSS}=\frac{\pi h{\sigma }_{f}}{2{\lambda }_{sat}}$$

In Eq. (1), *σ*_*f*_ is the fracture stress of the film, *λ*_*sat*_ is the average linear crack spacing at saturation and *h* is the film thickness. As stated previously, the fracture stress is best measured with XRD, however, many research groups have also used Hooke’s law and the observed fracture strain [36, 38, 47], while others included the residual stress of the film when known [48]. In order to properly apply the shear lag model to thin film fragmentation experiments, the ratio between the maximum and minimum crack spacings at saturation must be 2 [1, 47]. The ratio of 2 has been shown to change due to small changes in the film thickness or large surface roughnesses for brittle films on metal or polymer substrates [31, 49].

To calculate the Mode I fracture toughness,* K*_*Ic*_, of a brittle film, several models are available when the fracture stress is known [6, 14, 50–53]. The most promising model comes from Beuth [14] because it accounts for the elastic mismatch between film and substrate, which is taken into account by the Dundur’s parameters, *α* and *β* [54]. Using the steady state energy release rate, *G*_ss_, the fracture resistance of an individual layer can be evaluated with Eq. (2),

2 $$G_{{ss}} = \frac{{\pi \sigma _{f}^{2} h}}{{2E^{\prime}_{f} }}g\left( {\alpha ,\beta } \right)$$

where $E^{\prime}_{f} = E_{f} /\left( {1 - \nu _{f}^{2} } \right)$ with *E*_*f*_ is the elastic modulus of the film, *ν*_*f*_ is the Poisson’s ratio of the film, and $g\left(\alpha ,\beta \right)$ is a dimensionless parameter based on the Dundur’s parameters *α* and *β* [14, 54]. Finally, the apparent materials fracture toughness in terms of *K*_*Ic*_ is determined with the relationship: ${K}_{Ic}^{2}={G}_{ss}{E}_{f}{\prime}$, where the measured apparent fracture toughness *K*_app_ can be evaluated the same way for specific testing conditions. It should be noted that the experiment must be within the boundaries of linear-elastic fracture mechanics and with pure mode I loading to apply the above equations. Other thin film fracture toughness models are available, however only the above model will be showcased here.

## Results

**Fig. 2:**
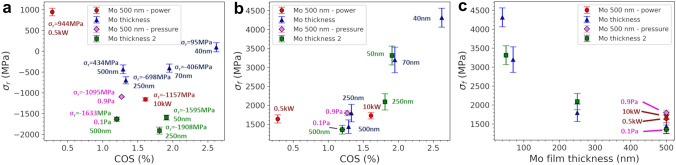
Comparison of **a** residual stress and COS of single Mo films illustrates that there is no significant trend. **b** The COS of various single Mo films increases with increasing fracture stress with film thickness having the largest influence. **c** Comparison of the fracture stress and film thickness for single layer Mo films also demonstrates the significance of the Mo film thickness on the measured fracture stress. Text next to each data point are the residual stresses and deposition power or film thickness

The residual and fracture stresses measured from the in-situ XRD experiments of the single Mo films are shown in Fig. 2 as a function of the COS and film thickness. It was observed that the COS increases with higher compressive residual stresses and thinner films (Fig. 2a), but that the fracture stress for the same film thickness remains relatively constant (Fig. 2b). Additionally, the thinner the film, the higher the fracture stress (Fig. 2c). The comparison shown in Fig. 2 is quite important as it demonstrates that even though the residual stress can increase the COS, the increased COS does not correlate to a higher fracture stress. If Hooke’s law were to be used for films with a higher COS due to increased compressive residual stresses, the real fracture stresses of the films would be significantly lower.

**Fig. 3:**
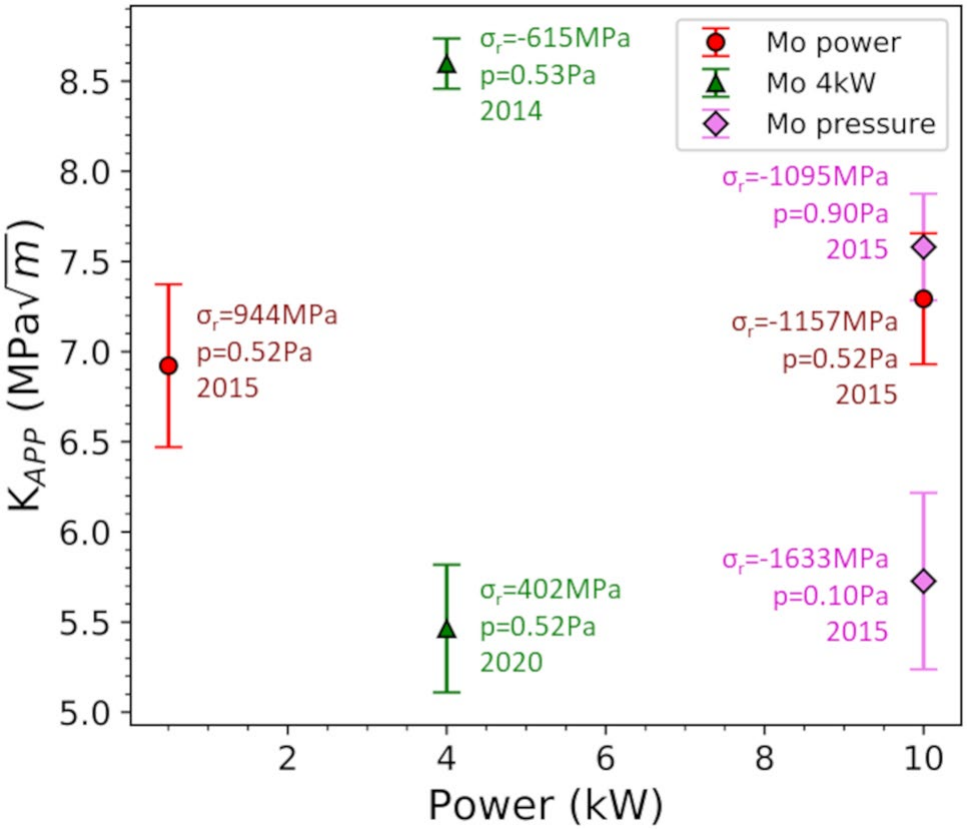
As the deposition power is varied, the *K*_*app*_ has no distinct trend. The highest *K*_*app*_ is measured for a film deposited with medium power resulting in a mildly compressive residual stress. Text next to each data point are the residual stresses, deposition power and year

The apparent fracture toughness of each film is evaluated with Eq. (2) and will be compared based on deposition parameters, film thickness, and architecture. When the sputter power for 500 nm thick films is compared (Fig. 3), large values are noted along with a lack of a trend. The values for *K*_*app*_ are near that found for bulk Mo-alloys [55, 56]. The error bars are calculated from the error of the fracture stress (error of fitting the Williamson-Hall plot—sin^2^*ψ*) and are important for a thorough and valid comparison of the different experiments. Note, each data point represents only one experiment where the residual stress of each data point with the respective deposition year are given. What one observes in Fig. 3 is that the residual stress does not contribute to the apparent fracture toughness, nor does the deposition power appear to matter. Two films deposited with the same parameters (power 4 kW) only 6 years apart illustrate that the same film cannot necessarily be created. The film deposited in 2014 had a compressive stress while the film deposited in 2020 had a tensile residual stress. For the three data points at 10 kW, all three have similar compressive residual stresses, but range in apparent fracture toughness between 5.7 and 7.6 MPa·m^1/2^ and were all deposited within the same year. The only open parameter is the microstructure of the films and from the initial work [32, 35] the crystallite domain size was measured to be 50 nm for the 0.5 kW film and 85 nm for the 10 kW film [33]. With so little difference between the lowest and highest powers, the microstructure is not expected to make a significant difference to the apparent fracture toughness. However, other factors like texture and point defect density have been shown to play an important role in determining the (nano)structure of thin films [33]. Further studies should be carried out to fully elucidate the role of micro- and nanostructure on thin film fracture behavior.

**Fig. 4:**
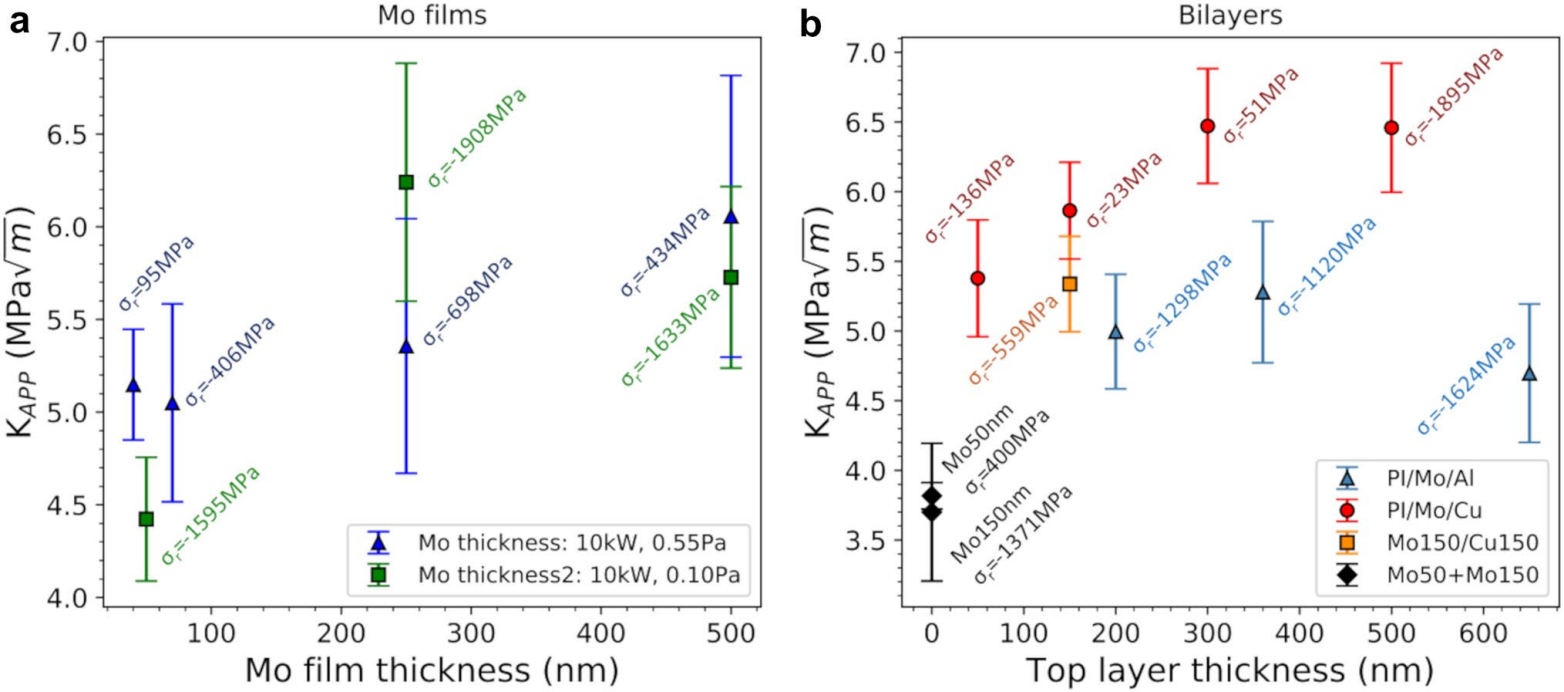
**a** Increasing film thickness influences the *K*_*app*_ more than the residual stress (text next to data points). **b** For Al/Mo and Cu/Mo bilayers of various thickness, the Mo interlayer *K*_*app*_ increases with increasing Al or Cu compared to the same single layer Mo thickness. Text next to each data point are the residual stresses and the error bars are determined from the accuracy of the XRD measurements

With no trends observed due to the power variation of the 500 nm Mo films, one could expect a trend with film thickness (Fig. 4a). A small increasing trend of the apparent fracture toughness as the film thickness increases is observed, but the magnitude of the increase is quite small. Again, each data point is one experiment with the error bars calculated from the error of the fracture stresses and the residual stresses are stated in the figure. Taking the thinnest films into account (less than 100 nm), the residual stresses range from almost stress free (95 MPa) to high compressive stresses (− 1.6 GPa). The 50 nm film with the lowest apparent fracture toughness has a very high compressive residual stress, which does not appear to be a benefit with regards to fracture toughness, only to the COS. As the film thicknesses are increased, the error bars become quite large and there is a small average increase from the thinner films. Again, the residual stress being compressive, to any magnitude, does not appear to significantly influence the apparent fracture toughness. The average crystallite domain size only ranged between 40 and 110 nm for film thicknesses of 40 to 500 nm [32, 35]. The factor of 3 difference in crystallite size over thickness range only has a minimal impact on the fracture behavior.

Finally, the influence of ductile overlayers are examined. It was recently shown for Cu/Mo bilayers that the apparent fracture toughness of the 50 nm Mo interlayers increased with increasing Cu film thickness [17]. To verify if the same trend was found for other ductile/brittle bilayers, the apparent fracture toughness of Al/Mo bilayers was calculated. As shown in Fig. 4b, the Mo interlayers in the Al/Mo bilayers exhibit the similar increasing trend with increasing ductile layer thickness. An additional Cu/Mo bilayer (150:150 nm) had the same trend. The increase in apparent fracture toughness is about 30–40% with the addition of a ductile overlayer. For both Cu and Al top layers, the critical film thickness ratio (ductile:brittle) is about 5:1, where any further increase in ductile film thickness either does not add any further benefit (i.e. Cu) or does not lead to any large changes in fracture toughness (i.e. Al). The single Mo films (in Fig. 4b at a thickness *x* = 0) have similar values for the different thicknesses and residual stresses, further illustrating that these film parameters are not crucial for the fracture toughness. It should be noted that more statistics are necessary to make more concrete statements, but the increasing trend is quite clear for the Mo interlayers.

## Discussion

**Fig. 5:**
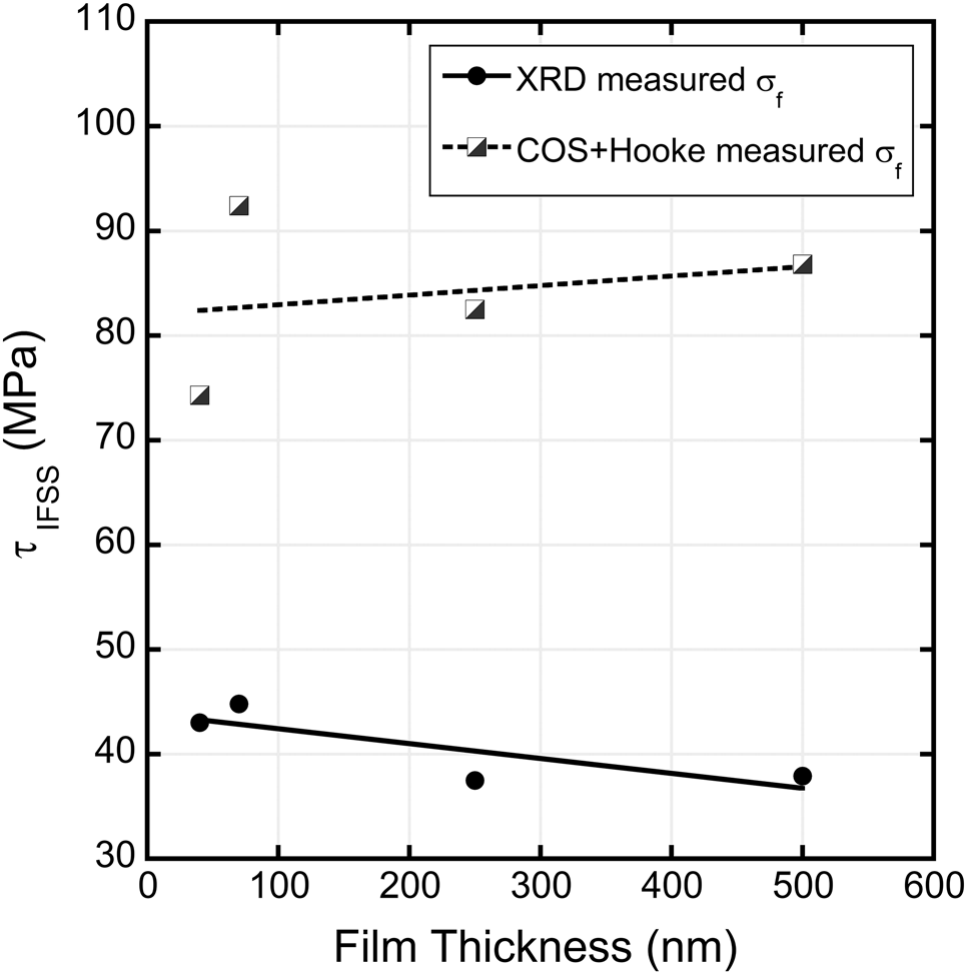
Comparison of interfacial shear stresses of single Mo films calculated using fracture stresses measured using XRD and COS with Hooke’s law. When calculated with XRD, the interfacial shear stresses are much lower and have a decreasing trend with thickness. Interfacial shear stress values calculated from the COS and Hooke’s law are significantly higher with an average increasing trend with thickness

From the differences in how to determine fracture stresses, variations in calculating the interfacial shear stresses or apparent fracture toughness would arise. To illustrate the difference, the experiments on single Mo films that vary in thickness were further examined [32]. Using Eq. (1), the interfacial shear stresses are directly compared in Fig. 5. When the apparent fracture stresses are estimated from the COS and Hooke’s law, the interfacial shear stresses are much higher than the more accurate values measured with XRD methods. The considerable difference between the two methods to measure the fracture stress should be a cause for concern. Using the COS to estimate the apparent fracture toughness via Hooke’s law leads to much higher values, especially when one takes the residual stresses into account. Residual stress tailoring is one method to increase the fracture strain of a single film [35] by introducing high compressive residual stresses. Compared to real fracture stresses measured with XRD, the apparent fracture stresses calculated with COS and Hooke’s law are almost double. Unless a correction can be performed, which would most likely not be universal for any film system, it is recommended to only use fracture stresses measured from XRD experiments for crystalline films and for amorphous films in-situ Raman could be used [57]. These values would be more accurate for the film behavior and take the residual stresses into account without the need for additional corrections. Tailoring the residual stresses will cause an offset in the COS that is not considered when estimating the fracture stress using Hooke’s law. One should take care when comparing interfacial shear stress or apparent fracture toughness values using fracture stresses measured from observed fracture strains.

The fact that only the film architecture influences the apparent fracture toughness should be considered significant. The simplest form of mode I fracture toughness from Griffith [58] is:

3 $${K}_{Ic}=\sigma \sqrt{\pi a}$$

with *a* being a crack length (thickness in the case of thin films) and *σ* the fracture stress. Considering the single Mo films, as the film thickness decreases, the fracture stresses increase at a similar rate, therefore, balancing out the effects of either parameter to have no significant trend.

When the microstructure of the films is considered for the examined films, again, no significant influence on the apparent fracture toughness was observed (Figs. 3 and 4). The microstructure would affect the measured stress–strain curves as well as the fracture and yield stresses. However, only using the crystallite domain size may not be sufficient for thorough analysis, also texture and point defect density can be expected to play a significant role. The investigated film crystallite domain size would be the smallest volume with a coherent crystal lattice and may not directly relate to a true grain size measured from transmission electron micrographs. Such micrographs would allow for a more thorough assessment, but are time and cost inefficient to examine every film in the present study. However, an unambiguous decoupling of the different effects of domain size, texture and point defect density on the fracture toughness will hardly be possible, since these effects are complexly intertwined in sputter deposition [59]. Therefore, reviewing the presented data, the most impactful parameter to *K*_*app*_ is the thickness of a ductile top layer, or in other words, the film architecture [17, 29].

## Conclusions

Variations of Mo thin films on Polyimide were examined using in-situ uniaxial tensile straining to determine what film parameters influence the fracture behavior. Film deposition parameters, film thickness, residual stresses, and film architecture were investigated. The fracture stresses were determined from in-situ straining experiments with XRD to get an accurate measurement of the true film fracture stresses. It was found that as the film thickness decreases for single Mo films, the fracture stresses measured with XRD increases. The fracture stress increase was also observed for Mo interlayers in bilayer film architectures with Cu or Al as the top layer. From the measured fracture stresses, the apparent fracture toughness was calculated with no noteworthy trends with regards to sputter power, film thickness, or residual stresses. Only the film architecture, especially the thickness of an overlying ductile layer, illustrated a meaningful trend with increasing apparent fracture toughness as thickness of the ductile layer increases. Additionally, fracture stresses calculated from the observed COS and Hooke’s law should be avoided as these stresses would be inaccurate since residual stress tailoring would increase or decrease the COS without much impact on the fracture stress, resulting in a strong over or under estimation of the apparent fracture toughness. The residual stress tailoring could also lead to different point defect densities in thin films, which could influence the fracture toughness. A comparison of fracture stresses measured with XRD and estimated with COS and Hooke’s law showed that the resulting interfacial shear stresses increase by about a factor of 2. Therefore, care should be taken when comparing to literature data. Finally, as there appears to be no thin film parameter that will impact the fracture toughness, a question that arises is what material or thin film parameter should be used when designing future flexible electronics or coatings that are more reliable.

## Data Availability

The data that support the findings of this study are available from the corresponding author upon reasonable request.
